# Cross Second Virial Coefficients of the H_2_O–H_2_ and H_2_S–H_2_ Systems from First-Principles

**DOI:** 10.1021/acs.jced.3c00300

**Published:** 2023-08-23

**Authors:** Robert Hellmann

**Affiliations:** Institut für Thermodynamik, Helmut-Schmidt-Universität/Universität der Bundeswehr Hamburg, Holstenhofweg 85, 22043 Hamburg, Germany

## Abstract

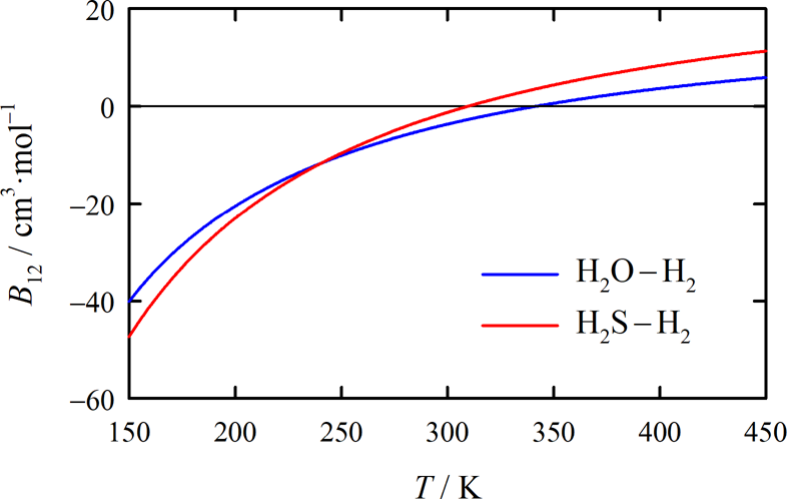

The cross second virial coefficients *B*_12_ for the interactions of water (H_2_O) with molecular hydrogen (H_2_) and of hydrogen sulfide (H_2_S) with H_2_ were obtained at temperatures in the range from 150 to 2000 K from new intermolecular potential energy surfaces (PESs) for the respective molecule pairs. The PESs are based on interaction energies determined for about 12 000 configurations of each molecule pair employing different high-level quantum-chemical ab initio methods up to coupled cluster with single, double, triple, and perturbative quadruple excitations [CCSDT(Q)]. Furthermore, the interaction energies were corrected for scalar relativistic effects. Both classical and semiclassical values for *B*_12_ were extracted from the PESs using the Mayer-sampling Monte Carlo approach. While our results for the H_2_O–H_2_ system validate the older first-principles results of Hodges et al. [J. Chem. Phys. **2004**, 120, 710–720], *B*_12_ for the H_2_S–H_2_ system was, to the best of our knowledge, hitherto neither measured experimentally nor predicted from first principles.

## 1 Introduction

The prediction of thermophysical properties of a molecular fluid requires detailed information about the interactions between the individual molecules. This information is contained in the so-called potential energy surface (PES), which is the function relating the positions, spatial orientations, and internal degrees of freedom of the molecules forming the fluid to the latter’s total potential energy. If we are dealing with a low-density gas and each molecule is approximated as a rigid rotor, we can formulate the PES as a sum of pair PESs, each of which is a function only of the separation and mutual orientation of the two interacting molecules. Quantum-chemical ab initio methods such as the popular coupled cluster approach with single, double, and perturbative triple excitations [CCSD(T)]^1^ can be applied to obtain very accurate interaction energies for pairs of small molecules that consist only of a few atoms. Once we have sampled the whole space of pair configurations sufficiently densely by such ab initio calculations, we can fit a suitable mathematical function to the calculated interaction energies to obtain the PES in analytical form.

An important thermophysical property that can be extracted readily both classically and semiclassically from rigid-rotor pair PESs is the second virial coefficient *B*, which determines the lowest-order correction to the ideal gas law in the virial expansion:
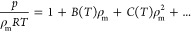
1where *p* is the pressure, ρ_m_ is the molar density, *R* is the molar gas constant, *T* is the temperature, and *C* is the third virial coefficient. *B* is a genuine two-body property and can be obtained as a function of *T* from statistical thermodynamics by numerically solving a multidimensional integral using a suitable integration algorithm. The virial expansion is also valid for gas mixtures. In a binary mixture with components 1 and 2, the total second virial coefficient *B*(*T*) is given through the exact relation
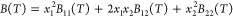
2where *x*_1_ and *x*_2_ are the mole fractions, *B*_11_ and *B*_22_ denote the corresponding second virial coefficients of the pure components, and *B*_12_ is the cross second virial coefficient (sometimes also called interaction second virial coefficient). Cross second virial coefficients can be extracted from the respective unlike-molecule pair PESs in a manner similar to that for the pure-component second virial coefficients from the like-molecule pair PESs.

In a number of recent studies, we applied the above-described first-principles methodology to predict cross second virial coefficients *B*_12_ for several pairs of small molecules, such as water (H_2_O) with nitrogen (N_2_), oxygen (O_2_), carbon monoxide (CO), carbon dioxide (CO_2_), hydrogen sulfide (H_2_S), and sulfur dioxide (SO_2_), see ref (2) and references therein, or H_2_S with N_2_^3^ and CO_2_.^4^ The present work extends our series of first-principles investigations on cross second virial coefficients to two systems containing molecular hydrogen (H_2_), namely, H_2_O–H_2_ and H_2_S–H_2_. Reliable thermophysical property data for these systems are important for the hydrogen economy. For example, data for the H_2_O–H_2_ system are needed for modeling combustion in hydrogen gas turbines. In other applications, small amounts of H_2_O are present in H_2_ gas as an impurity whose effects need to be accurately modeled. Property data for H_2_S–H_2_ mixtures are also of interest since H_2_S separated from sour natural gas is a potential source for producing H_2_ through decomposition. Ab initio PESs for both systems (and for H_2_O–H_2_ also derived *B*_12_ values) already exist, see, e.g., refs (5−10). However, as in our previous studies on cross second virial coefficients, we developed new pair PESs, applying higher levels of theory for the ab initio calculations. The PES models used in this work are summarized in Table 1. We stress again that the respective pure-component PESs (see, e.g., refs (11−13) for ab initio PESs from the literature) are not required for the calculation of the cross second virial coefficients.

**Table 1:** PES Models Used in This Work

chemical name	chemical formula	CAS number	type of PES	specific PES model
water	H_2_O	7732-18-5	intramolecular	rigid-rotor approximation
hydrogen sulfide	H_2_S	7783-06-4	intramolecular	rigid-rotor approximation
molecular hydrogen	H_2_	1333-74-0	intramolecular	rigid-rotor approximation
water-hydrogen molecule pair	H_2_O–H_2_		intermolecular	new PES of this work
hydrogen sulfide-hydrogen molecule pair	H_2_S–H_2_		intermolecular	new PES of this work

The paper is organized as follows: In section 2, we present the new ab initio pair PESs. The details of the calculations of *B*_12_ in the temperature range from 150 to 2000 K are provided in section 3. In section 4, we present the results for *B*_12_ both as a data table of discrete values and as practical correlations. We also compare our results with the available experimental and first-principles-based values from the literature for *B*_12_ and the related dilute gas cross isothermal Joule–Thomson coefficient ϕ_12_. Finally, our conclusions are given in section 5.

## 2 H_2_O–H_2_ and H_2_S–H_2_ Potential Energy Surfaces

### 2.1 Monomer Geometries and Multipole Moments

In this work, the monomers H_2_O, H_2_S, and H_2_ were approximated as rigid rotors, meaning that all of the bond lengths and bond angles were kept at fixed values. The resulting reduction in the dimensionality of the H_2_O–H_2_ and H_2_S–H_2_ PESs from 9 to 5 comes at the expense of a somewhat lower (but still very good) accuracy for the derived *B*_12_ values. The equilibrium structures (i.e., the minima of the intramolecular PESs as obtained by standard geometry optimizations) are not an optimal choice for the fixed monomer geometries. Instead, the zero-point vibrationally averaged structures are recommended to be used^14^ because they at least partly account for the effects of the suppressed degrees of freedom. The respective H_2_O geometry was taken from ref (14) and is characterized by an OH bond length of 0.9716257 Å (1 Å = 10^–10^ m) and an HOH angle of 104.69°. The averaged H_2_S geometry was taken from our work on the H_2_S–H_2_S system^13^ and is characterized by an SH bond length of 1.3506 Å and an HSH angle of 92.219°, while for the averaged bond length of the H_2_ molecule a value of 0.766638 Å^15,16^ was adopted.

Electric multipole moments for the three monomers were also determined for the purpose of using them as constraints in the fits of the electrostatic parts of the analytical potential functions (see section 2.3). For H_2_, we determined the quadruple moment at the averaged bond length by interpolating the separation-dependent quadrupole moment values calculated by Komasa and Thakkar,^17^ yielding a value of 0.482390 au. In our recent work on the H_2_O–H_2_S and H_2_O–SO_2_ systems,^2^ we already determined values for the dipole moments and for the components of the quadrupole and octopole moment tensors of H_2_O and H_2_S at the CCSD(T)^1^ level of theory with scalar relativistic corrections, and we do not repeat the details of these calculations here. However, because the highest level of theory used for the present ab initio calculations of H_2_O–H_2_ and H_2_S–H_2_ interaction energies is higher than CCSD(T), it is appropriate to also include a post-CCSD(T) correction for the multipole moments of H_2_O and H_2_S for consistency. These corrections were obtained as the differences between the respective multipole moment values obtained at the frozen-core (FC) CCSDTQ^18,19^ and CCSD(T) levels of theory using the aug-cc-pVTZ^20−22^ basis set with the analytical derivative approach implemented in the CFOUR^23,24^ quantum chemistry code (version 2.1) with orbital relaxation included. Because the required derivatives cannot be computed at the CCSDTQ level by the employed CFOUR version, the general coupled cluster code MRCC^25,26^ (2020 version) interfaced with CFOUR was used for these calculations. The relative changes in the multipole moment values due to the post-CCSD(T) correction were found to be very small, being within ±0.23% for both molecules. The details are provided in the Supporting Information.

### 2.2 Pair Geometries and Interaction Energies

**Figure 1:**
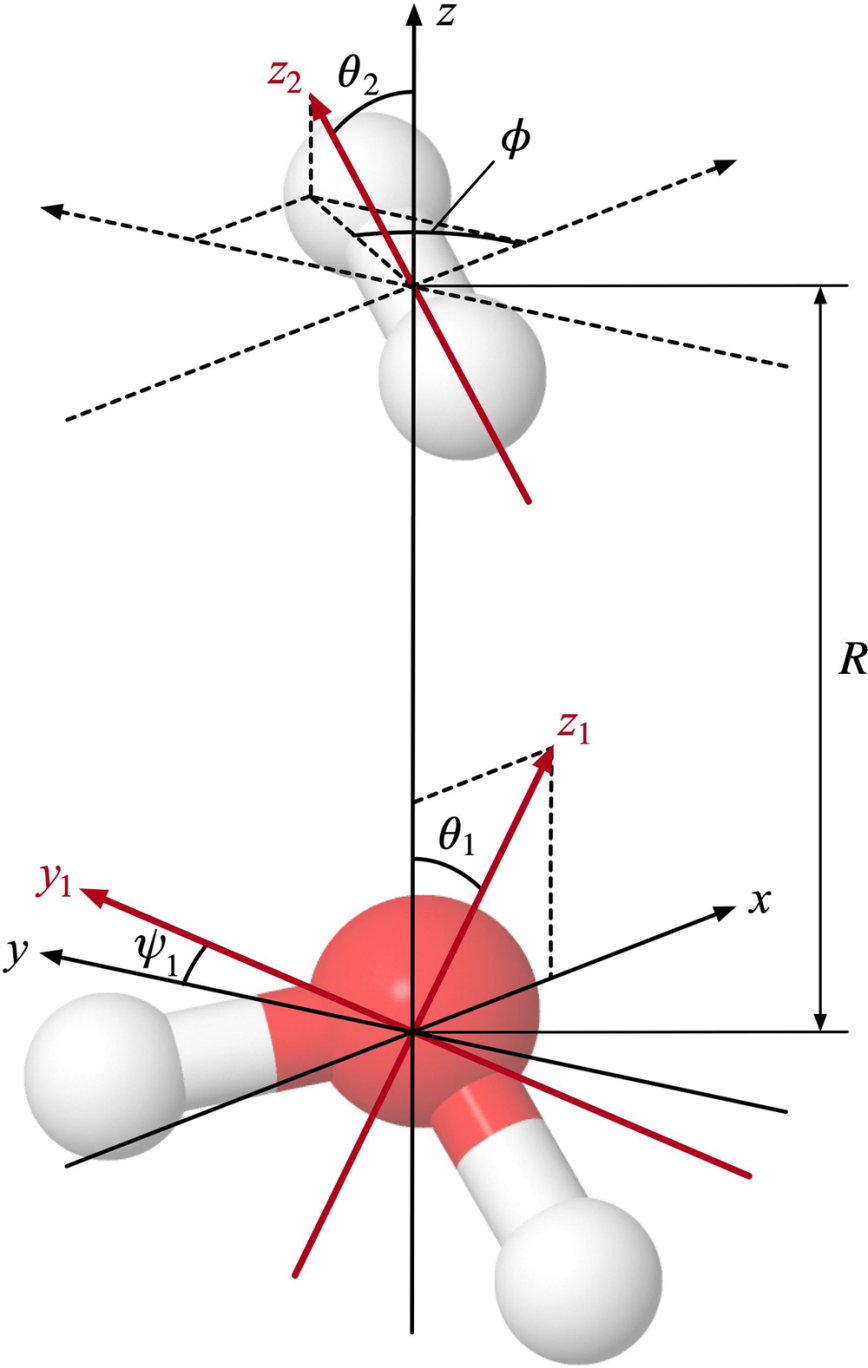
Visualization of the variables *R*, θ_1_, ψ_1_, θ_2_, and ϕ used to describe H_2_O–H_2_ and, in an analogous manner, H_2_S–H_2_ configurations. The center of mass of the H_2_O molecule is located at the origin of a Cartesian coordinate system defined by the axes *x*, *y*, and *z*, while the H_2_ molecule’s center of mass is at (*x*, *y*, *z*) = (0, 0, *R*). The molecule-fixed axes *z*_1_ and *z*_2_ are the symmetry axis of H_2_O and the bond axis of H_2_, respectively. The molecule-fixed axis *y*_1_ lies in the molecular plane of H_2_O and is perpendicular to *z*_1_; it crosses *z*_1_ in the center of mass of the H_2_O molecule, i.e., at (*x*, *y*, *z*) = (0, 0, 0).

The geometries of the systems H_2_O–H_2_ and H_2_S–H_2_ within the rigid-rotor approximation can be described by the separation between the centers of mass of the two molecules, *R*, and the four Euler angles θ_1_, ψ_1_, θ_2_, and ϕ. This is illustrated exemplarily for the H_2_O–H_2_ system in Figure 1, with further details given in the Supporting Information. To exhaustively sample the angular configuration space for both systems, we systematically varied θ_1_ in steps of 22.5° starting with 0°, ψ_1_ and θ_2_ in steps of 30° starting with 0°, and ϕ in steps of 45° starting with 0°. The resulting set of angular configurations was checked for configurations that are related to one another through symmetry operations and are, thus, energetically equivalent. By discarding the redundant configurations, we obtained 510 unique angular configurations. For each angular configuration, we considered 24 center-of-mass separations *R* in the range from 1.2 to 10.0 Å, yielding 12 240 (510 × 24) pair configurations. Some configurations at very small separations were discarded immediately because of excessive overlap of the two molecules, while other configurations, also at small separations, had to be discarded because of unresolvable convergence problems in the quantum-chemical calculations. The number of remaining pair configurations was 12 097 for the H_2_O–H_2_ system and 12 092 for the H_2_S–H_2_ system.

We used the counterpoise-corrected supermolecular approach^27^ to calculate the interaction energies *V* for all pair configurations. An additivity scheme combining different levels of theory was employed in order to achieve the highest possible accuracy for the final interaction energies. As the lowest level of theory, for which the largest basis sets could be used, we chose the resolution of identity second-order Møller–Plesset perturbation theory (RI-MP2)^28,29^ within the FC approximation, with the RI-JK approximation^30,31^ applied in the Hartree–Fock (HF) step. The calculations were carried out using the aug-cc-pV*X*Z^20−22^ basis sets with *X* = 4 (Q) and *X* = 5 together with the auxiliary basis sets aug-cc-pV5Z-JKFIT^32^ and aug-cc-pV5Z-MP2FIT^33^ for both values of *X*. Following Patkowski,^34^ we also placed a “ghost” hydrogen atom with the same basis and auxiliary basis functions as for the real hydrogen atoms midway along the *R* axis of each pair configuration because such so-called bond functions usually significantly accelerate convergence toward the complete basis set (CBS) limit. The correlation contributions to the computed interaction energies, *V*_RI–MP2 corr_^*X*^, were extrapolated to the CBS limit with the widely used *X*^–3^ scheme:^35,36^
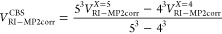
3whereas the HF contributions were taken from the calculations at the *X* = 5 basis set level without extrapolation.

In the next step, we calculated the differences between the CCSD(T)^1^ and the standard MP2 interaction energies within the FC approximation using the aug-cc-pV*X*Z^20−22^ basis sets with *X* = 3 (T) and *X* = 4 (in both cases without bond functions). The differences, denoted as Δ*V*_CCSD(T)_^*X*^, were extrapolated to the CBS limit again using the *X*^–3^ scheme:
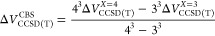
4We then added Δ*V*_CCSD(T)_^CBS^ to the total RI-MP2 interaction energies, yielding values that should be very close (probably within 1% for most orientations in the well regions of the PESs) to the true CBS limits of the total CCSD(T) interaction energies within the FC approximation.

Usually, CCSD(T) or a variant thereof is the highest level of theory applied in the development of intermolecular PESs, and this is also the case for the existing PESs for the H_2_O–H_2_ and H_2_S–H_2_ systems.^5−7,9,10^ However, because the H_2_ molecule has only two electrons, the total number of electrons in the H_2_O–H_2_ and H_2_S–H_2_ molecule pairs is small enough to make calculations at still higher levels of theory, in particular CCSDT(Q),^37,38^ with reasonably sized basis sets computationally feasible today. We calculated the CCSDT(Q) correction for all configurations by taking the differences between the interaction energies obtained at the CCSDT(Q) and CCSD(T) levels within the FC approximation employing the aug-cc-pV*X*Z^20−22^ basis sets with *X* = 2 (D) and *X* = 3, followed again by extrapolation to the CBS limit:
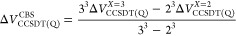
5For both molecule pairs, Δ*V*_CCSDT(Q)_^CBS^ is found to be always negative. Its relative influence on the total interaction energy exceeds 1%, 2%, and 4% for about 48%, 29%, and 17%, respectively, of all H_2_O–H_2_ configurations and for about 61%, 37%, and 17%, respectively, of all H_2_S–H_2_ configurations.

Finally, corrections were calculated for the inclusion of core–core and core–valence correlations, Δ*V*_core_, and scalar relativistic effects, Δ*V*_rel_. We determined Δ*V*_core_ at the CCSD(T)/aug-cc-pwCVTZ^20−22,39^ level by computing the differences between the interaction energies obtained with all but the sulfur 1*s* electrons correlated and the interaction energies resulting with the FC approximation. The relativistic corrections Δ*V*_rel_ were determined at the CCSD(T) level within the FC approximation using the aug-cc-pVTZ basis set in uncontracted form by taking the differences between the interaction energies resulting with the spin-free exact two-component theory at the one-electron level^40^ and the nonrelativistic interaction energies. The combined effect of Δ*V*_core_ and Δ*V*_rel_ on the total H_2_O–H_2_ interaction energies is very small and essentially negligible. For half of all investigated configurations, the impact is less than 0.15%, while it is higher than 1% for only about 5% of the configurations. Not surprisingly, the two corrections are more important for the H_2_S–H_2_ system; their combined impact on the interaction energy exceeds 1%, 2%, and 4% for about 42%, 21%, and 10%, respectively, of the investigated configurations.

Detailed results of the ab initio calculations for all 12 097 H_2_O–H_2_ and 12 092 H_2_S–H_2_ configurations are provided in the Supporting Information. The ORCA^41,42^ program (version 3.0.3) was used to perform the RI-MP2 calculations, while all other calculations were carried out with CFOUR^23,24^ (version 2.1).

### 2.3 Analytical Potential Functions

The two new pair PESs are represented analytically by site–site functions. We chose an arrangement with nine sites for both the H_2_O and H_2_S molecules. All of the sites are located in the molecular planes, and in both molecules, three of the nine sites are located directly on the *C*_2*v*_ symmetry axis. For the smaller H_2_ molecule, five interaction sites were deemed sufficient. With these site arrangements, due to symmetry, H_2_O and H_2_S each only have six distinct types of sites, while H_2_ only has three distinct types of sites. The number of distinct types of site–site interactions and the total number of site–site interactions in each molecule pair are thus 18 (6 × 3) and 45 (9 × 5), respectively. We represent each of the site–site interactions by the following function:
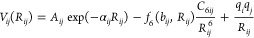
6where *R*_*ij*_ is the separation between site *i* in H_2_O or H_2_S and site *j* in H_2_, and the damping functions *f*_6_ are those of Tang and Toennies:^43^
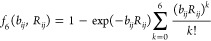
7The total pair potential is obtained by summing over all site–site contributions:
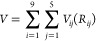
8

We determined the parameters *A*, α, *b*, and *C*_6_ for the distinct types of site–site combinations (72 parameters in total per molecule pair), the precise locations of the sites in the molecules, and the site charges *q* (fixed at zero for one of the H_2_O sites) by performing nonlinear least-squares fits to the 12 097 H_2_O–H_2_ and 12 092 H_2_S–H_2_ ab initio interaction energies. The fits were constrained such that the molecules have zero charge and that the ab initio multipole moments of the monomers (see section 2.1) are reproduced. Furthermore, to ensure that the fit quality is appropriately balanced over the entire PESs, the ab initio interaction energies were weighted with the following function:
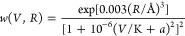
9where *a* = 350 for the H_2_O–H_2_ PES and *a* = 220 for the H_2_S–H_2_ PES. The denominator in eq 9 increases the weight of configurations as the interaction energy decreases toward the most negative values (*V* > −350 K and *V* > −220 K for all investigated H_2_O–H_2_ and H_2_S–H_2_ configurations, respectively), whereas the numerator ensures that the fit quality does not seriously deteriorate at large *R* values. We used similar weighting functions previously (e.g., in ref (2)). Note that throughout this work, we quote energies in units of kelvin; i.e., we divided the energies by the Boltzmann constant *k*_B_. However, we omit *k*_B_ from the notation for brevity.

**Figure 2:**
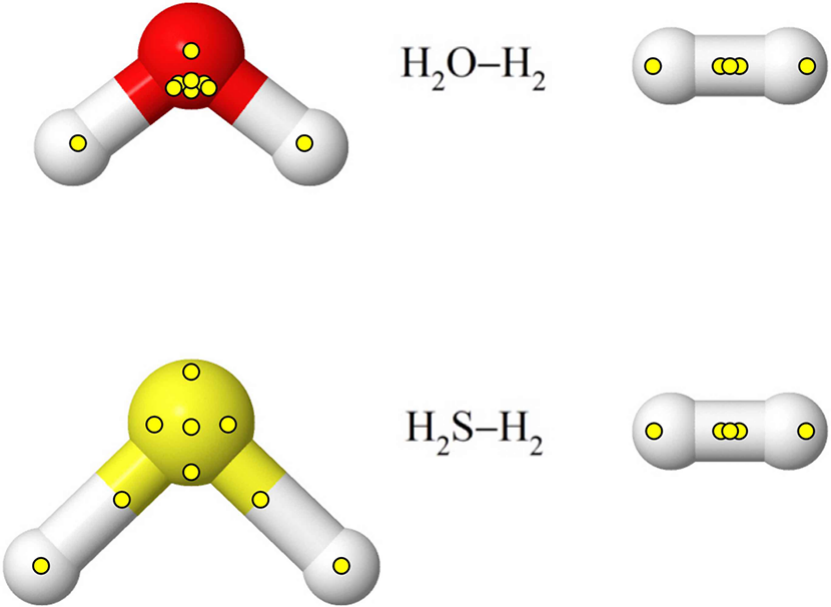
Optimized positions of the nine H_2_O, nine H_2_S, and five H_2_ interaction sites for the two potential functions. The sites are all located in the molecular planes.

Figure 2 visualizes the positions of the interaction sites relative to the atoms in the molecules for both potential functions. Although barely discernible, the positions of the five sites in H_2_ are different for the H_2_O–H_2_ and H_2_S–H_2_ potential functions.

**Figure 3:**
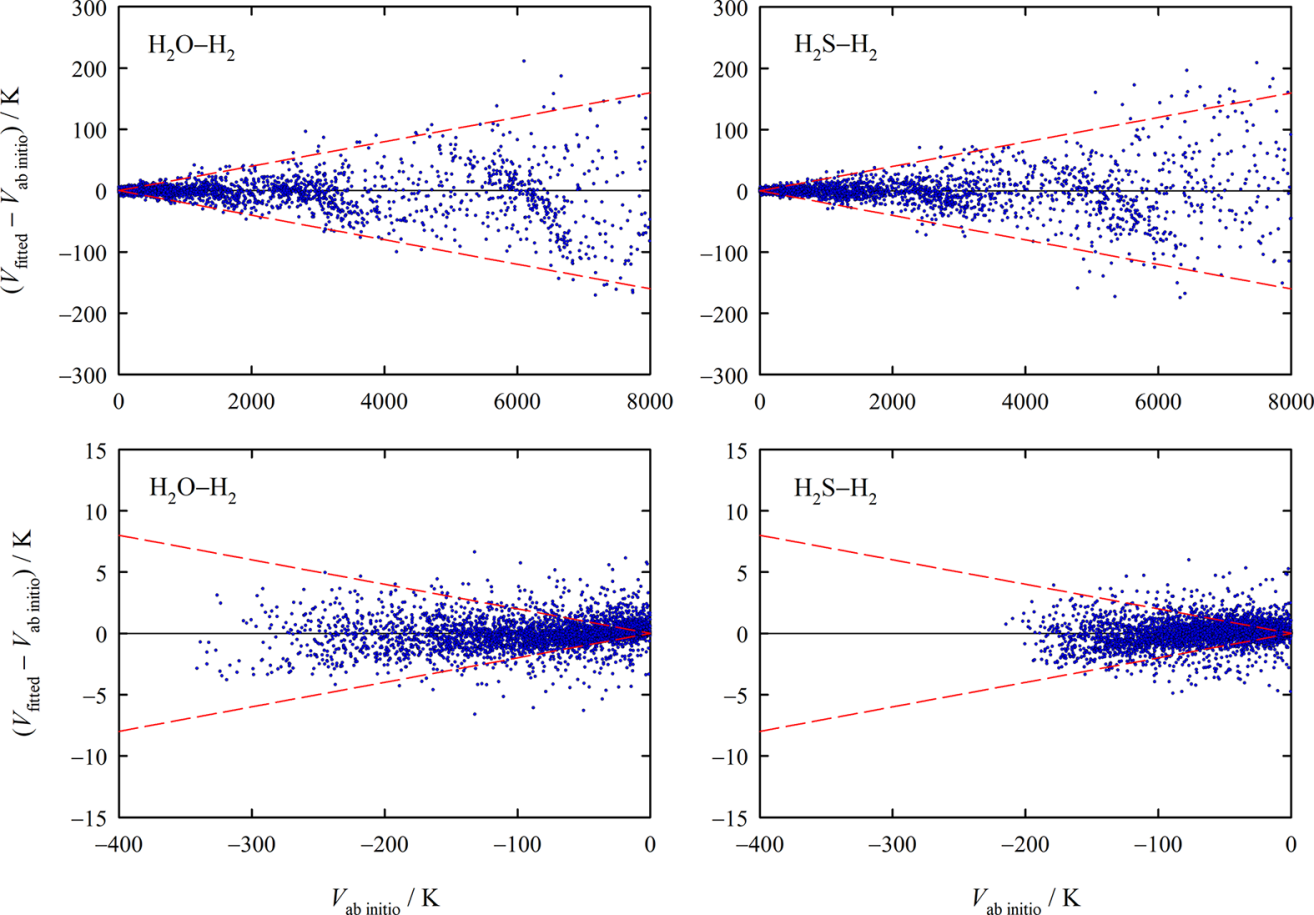
Deviations of interaction energies obtained using the new potential functions from the corresponding ab initio calculated interaction energies as a function of the latter for positive interaction energies up to 8000 K (upper panels) and negative interaction energies (lower panels). The dashed red lines are a guide to the eye indicating relative deviations of ±2%.

Figure 3 depicts the deviations of the interaction energies obtained with the fitted potential functions from the corresponding ab initio calculated ones. With the relative deviations being mostly within ±2%, the fit quality can be considered as sufficient for accurate calculations of thermophysical properties because the effects on the calculated property values by fitting errors with positive and negative sign are expected to mostly cancel out. We restricted Figure 3 to interaction energies lower than 8000 K because higher energies are of quickly diminishing relevance for the present applications of the potential functions. For both PESs, the full range of ab initio interaction energies extends to more than 160 000 K. A visual inspection of many one-dimensional cuts through the potential functions along *R* and the four angles showed that the functions are very smooth; i.e., there are no apparent unphysical “wiggles,” which can occur when a function with too many adjustable parameters is fitted to too few ab initio points. While the potential functions, due to the chosen form and applied constraints, behave correctly at *R* values above the investigated range, the behavior of the potential functions at the very small *R* values below the investigated range is partly unphysical. This is manifested by the occurrence of spurious saddle points for some angular configurations, so that *V* actually decreases as *R* is further decreased, often to the point that *V* becomes negative. Such a “hole” is problematic if the saddle point separating it from the rest of the PES is thermally accessible at the temperatures at which we calculate the thermophysical properties. Fortunately, even at the highest temperature investigated in this work (2000 K), the lowest of these spurious saddle points with interaction energies of about 95 000 K (H_2_O–H_2_) and 73 000 K (H_2_S–H_2_) are still completely inaccessible. As described in section 3, the holes can be relatively easily addressed in this case.

**Figure 4:**
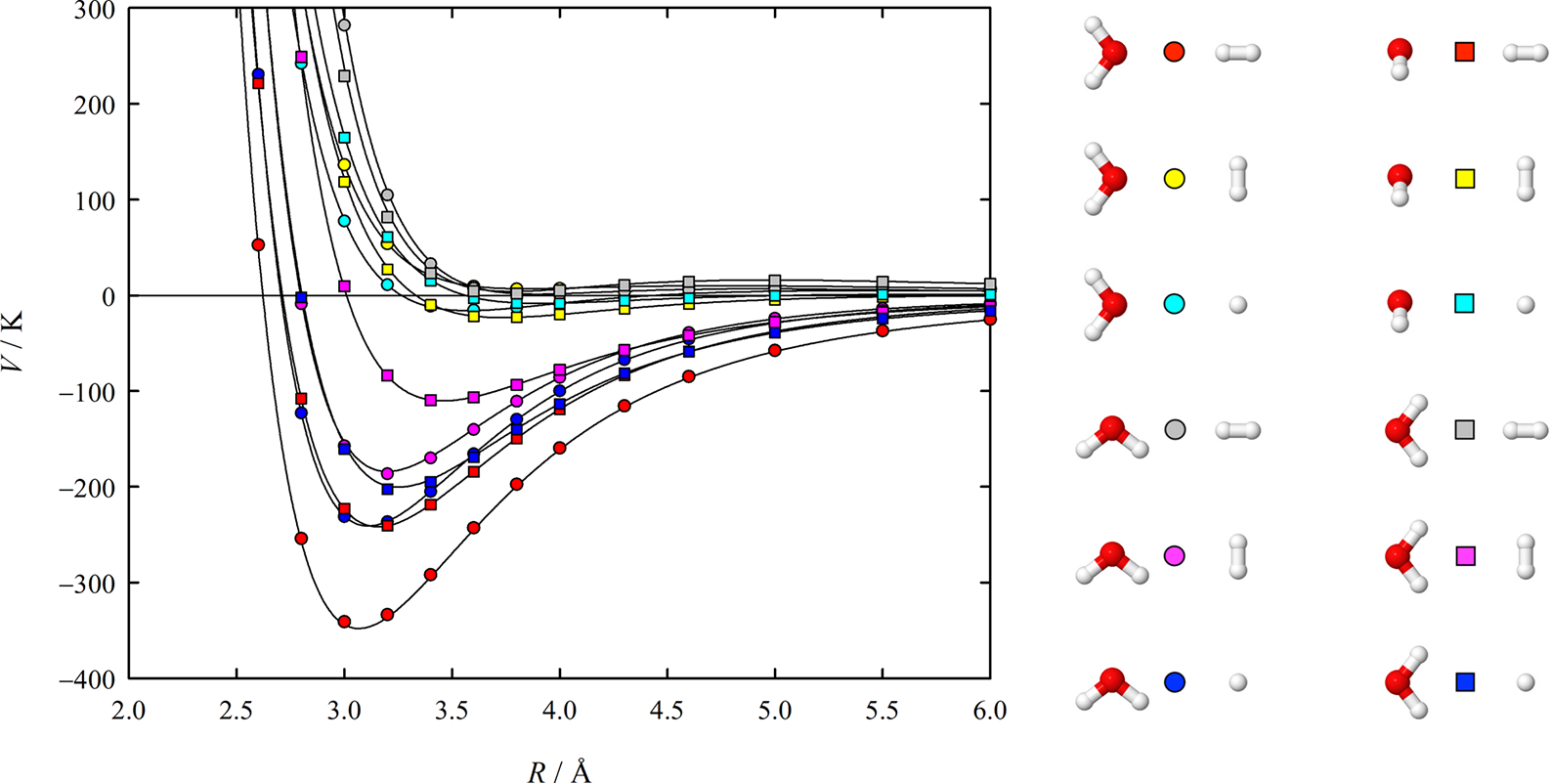
H_2_O–H_2_ interaction energies as a function of the center-of-mass separation *R* for 12 of the 510 investigated angular configurations. Symbols indicate the discrete ab initio interaction energies, while solid lines indicate the interaction energies obtained for the respective angular orientations with the fitted potential function.

**Figure 5:**
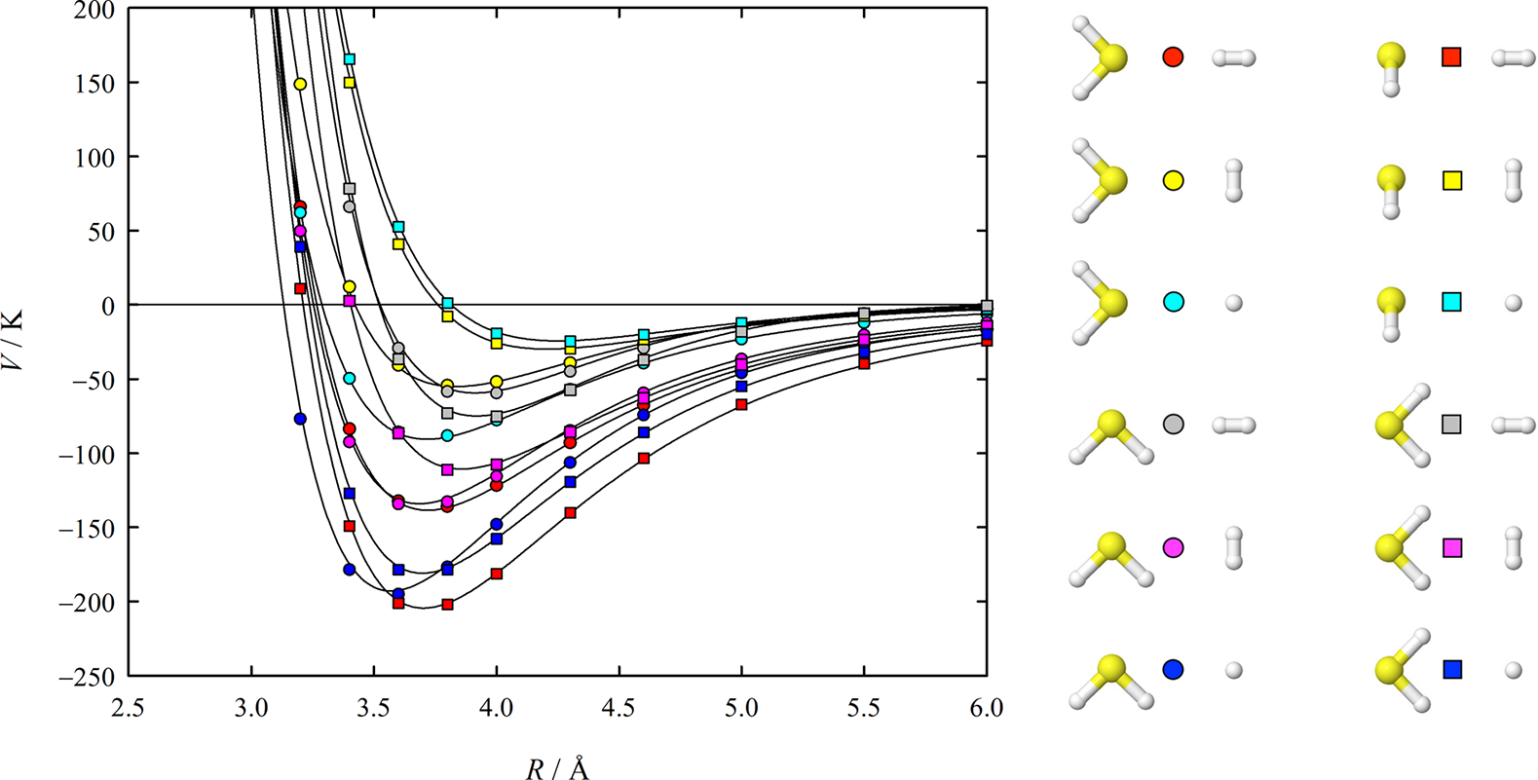
H_2_S–H_2_ interaction energies as a function of the center-of-mass separation *R* for 12 of the 510 investigated angular orientations. The meaning of the symbols and lines is the same as that in Figure 4.

Figures 4 and 5 show cuts along *R* through the H_2_O–H_2_ and H_2_S–H_2_ potential functions, respectively, for 12 selected angular configurations. The figures demonstrate that the anisotropy of the pair interactions is especially strong with respect to the well depth.

**Figure 6:**
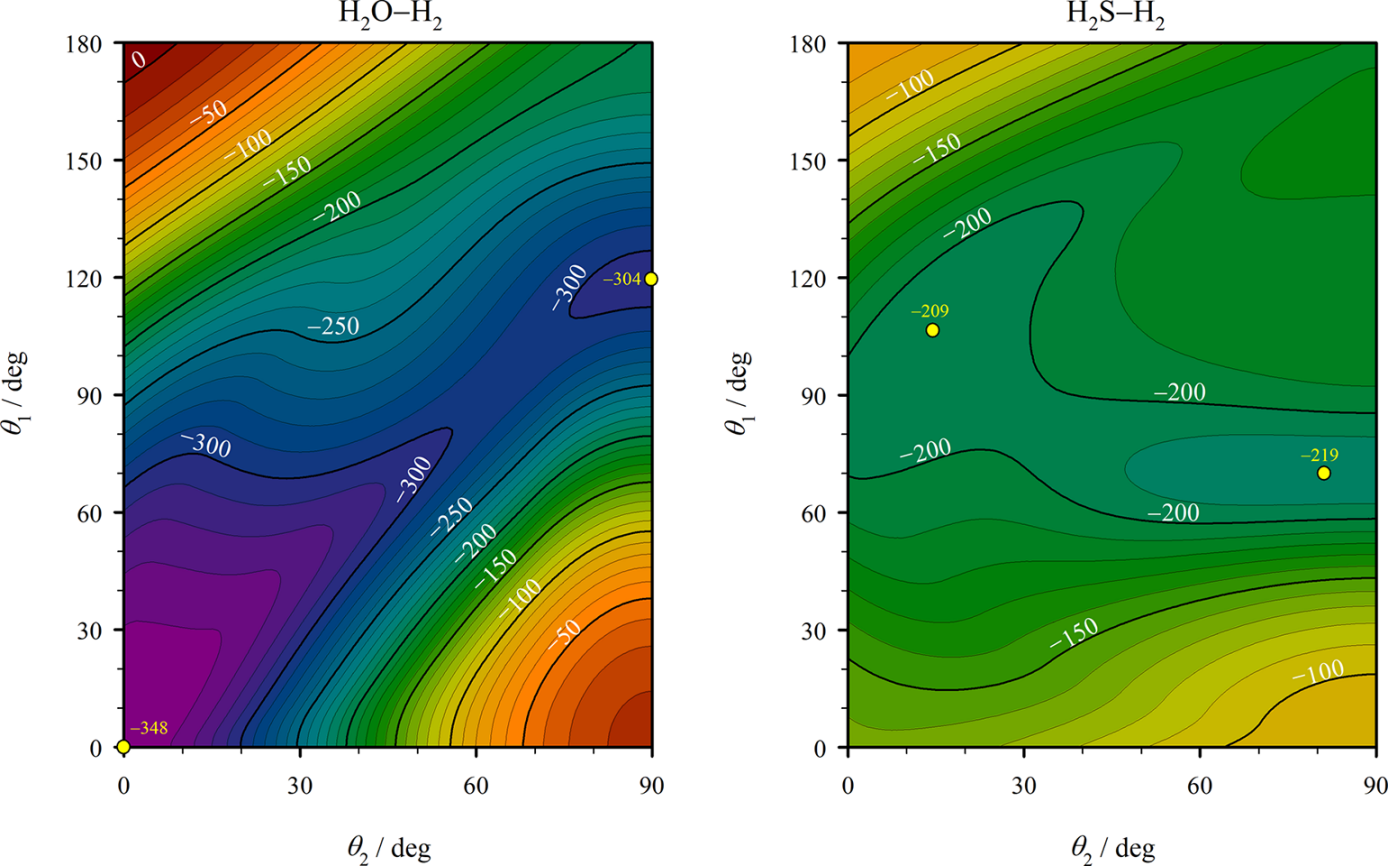
Contour plots of *V* (in kelvin) for the analytical H_2_O–H_2_ and H_2_S–H_2_ PESs of this work as a function of the angles θ_1_ and θ_2_, with the center-of-mass separation *R* and the angles ψ_1_ and ϕ chosen such that *V* is minimized for each combination of θ_1_ and θ_2_. The minima of the PESs (apart from the dubious third minimum of the H_2_S–H_2_ PES) are marked in the plots.

The H_2_O–H_2_ potential function features two energetically distinct minima, which are characterized by interaction energies of about −348 K and −304 K. The value for the global minimum is in excellent agreement with the respective value given by Hodges et al.^5^ for their PES of about −346 K, whereas the global minimum of the widely used PES of Valiron et al.^7^ is distinctly shallower with an interaction energy of about −339 K. Valiron et al. also provided a value for the interaction energy of the secondary minimum, about −291 K, which is again significantly less negative than our value of −304 K, whereas Hodges et al. did not quote the respective energy for their PES. Our H_2_S–H_2_ potential function features three energetically distinct minima, which are characterized by interaction energies of about −219 K, –209 K, and −182 K. However, the −182 K minimum lies only about 0.03 K below an adjacent saddle point, so that it could as well be just a fitting artifact. The only other ab initio H_2_S–H_2_ potential function we are aware of is that of Dagdigian,^10^ who found also three minima and gives values of about −211 K, −197 K, and −176 K for their interaction energies. While the first two values can be directly compared with our distinctly more negative values of −219 K and −209 K, Dagdigian’s −176 K minimum has a structure that corresponds to a saddle point on our PES and is quite different from our disputable −182 K minimum. To provide a graphical illustration of the locations of the minima on the H_2_O–H_2_ and H_2_S–H_2_ PESs of the present work, Figure 6 shows contour plots of *V* as a function of the angles θ_1_ and θ_2_, with the remaining three variables chosen such that *V* is minimized. It can be seen that for both PESs the saddle point separating the global minimum and the secondary minimum lies energetically only slightly above the secondary minimum. The full details regarding the minima of the potential functions are given in the Supporting Information. Fortran 90 programs that compute the potential functions are also provided there.

## 3 Calculation of the Cross Second Virial Coefficients

Classical statistical thermodynamics provides a relatively simple expression for the cross second virial coefficient of a pair of rigid molecules:
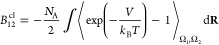
10where *N*_A_ is Avogadro’s constant, **R** is the separation vector between the two molecules, and the angle brackets indicate proper averaging over the spatial orientations of the two molecules, which we represent by the variables Ω_1_ and Ω_2_.

Particularly due to the H_2_ molecule’s very small mass and moment of inertia, it is essential to account for nuclear quantum effects, which in this work we did with two well-established semiclassical schemes. The quadratic Feynman–Hibbs (QFH) approach^44^ adds a temperature-dependent term to the potential *V*:
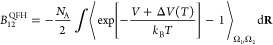
11where Δ*V*(*T*) is given for the present case of molecule 1 (H_2_O or H_2_S) being an asymmetric top and molecule 2 (H_2_) being linear as
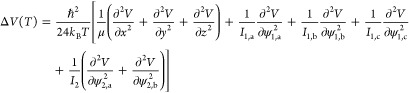
12Here, *ℏ* is Planck’s constant *h* divided by 2π; μ denotes the reduced mass of the molecule pair; *x*, *y*, and *z* are the Cartesian components of **R**; *I*_1,a_, *I*_1,b_, and *I*_1,c_ are the three principal moments of inertia of molecule 1; *I*_2_ is the moment of inertia of molecule 2 perpendicular to its bond axis; and the angles ψ_1,a_, ψ_1,b_, ψ_1,c_, ψ_2,a_, and ψ_2,b_ describe rotations around the principal axes of the molecules with the exception of the bond axis of molecule 2. The second semiclassical approach that we considered differs from the QFH approach in that it does not modify the potential *V* but instead modifies the classical expression by introducing its first-order quantum correction:
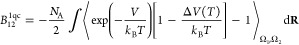
13where Δ*V*(*T*) has the same form as for the QFH approach, although it is possible to simplify it in this case through integration by parts such that it involves only first derivatives. Both the QFH approach and the first-order quantum correction approach (which we refer to as 1qc approach) are correct only up to order *ℏ*^2^ with respect to the complete semiclassical expansion of *B*_12_ in powers of *ℏ*^2^. While apparently *B*_12_^1qc^ does not contain any contributions of orders *ℏ*^4^, *ℏ*^6^, and so forth, *B*_12_^QFH^ does due to Δ*V*(*T*) appearing inside the exponential. These higher-order contributions can be viewed as extremely crude estimates of the true higher-order quantum corrections, so that in most cases, *B*_12_^QFH^ can be expected to be closer to the fully quantum-mechanical result than *B*_12_^1qc^.

To evaluate *B*_12_ numerically for both molecule pairs at the classical and the two semiclassical levels of theory, we used the Mayer-sampling Monte Carlo (MSMC) approach devised by Singh and Kofke.^45^ The calculations were performed with the same in-house code as in all of our previous studies on cross second virial coefficients. During the MSMC runs, *B*_12_ averages were accumulated simultaneously for 99 temperatures from 150 to 2000 K by applying the multitemperature variant of the MSMC approach,^46^ with the temperature governing the sampling distribution being 300 K. To avoid sampling of the holes in the PESs at very small separations, we placed hard spheres with a diameter of 1 Å on all interaction sites in the molecules and checked that the spheres are large enough to cover the holes completely but not so large that their presence introduces systematic errors into the computed *B*_12_ values. Each attempted MSMC move consisted of a random displacement and rotation of one of the two molecules, which was also chosen randomly. The maximum step sizes were iteratively adjusted in short equilibration runs until an acceptance rate of close to 50% was achieved. The derivatives of the pair PESs needed for the semiclassical schemes were computed analytically. As the reference system required for the MSMC approach, we used simple hard spheres of 4.5 Å diameter placed at the centers of mass of the molecules. For each molecule pair and level of theory, 16 independent simulation runs, each comprising 5 × 10^10^ attempted moves, were carried out, and the resulting *B*_12_ values for each temperature were averaged. The statistical standard uncertainties of the final *B*_12_ values due to the integration are always smaller than 0.001 cm^3^·mol^–1^ and are thus so small that they are irrelevant to the overall uncertainty assessment.

## 4 Results and Discussion

Table 2 lists the values for the cross second virial coefficients obtained with the new pair PESs at the classical and the semiclassical 1qc and QFH levels of theory for the H_2_O–H_2_ and H_2_S–H_2_ systems at 47 of the 99 investigated temperatures. In addition, the table provides values for a “tuned” QFH level for the H_2_O–H_2_ system:
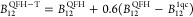
14where the factor 0.6 is the result of approximately matching the differences between *B*_12_^QFH–T^ and *B*_12_^1qc^ at 150, 200, and 250 K with the differences between the fully quantum-mechanically calculated *B*_12_ values of Hodges et al.^5^ and their respective 1qc values at these temperatures. This tuning approach is particularly justified because the first-order quantum corrections obtained in this work and by Hodges et al. agree almost perfectly.

**Table 2:** Cross Second Virial Coefficients *B*_12_ (in cm^3^·mol^–1^) of the H_2_O–H_2_ and H_2_S–H_2_ Molecule Pairs at Selected Temperatures *T* at the Classical, the Semiclassical 1qc, the Semiclassical QFH, and, for H_2_O–H_2_, the “Tuned” Semiclassical QFH Levels of Theory (*B*_12_^cl^, *B*_12_^1qc^, *B*_12_^QFH^, and *B*_12_^QFH–T^, Respectively) as Well as the Estimated Combined Expanded (*k* = 2) Uncertainties *U*(*B*_12_^QFH–T^) for H_2_O–H_2_ and *U*(*B*_12_^QFH^) for H_2_S–H_2_

	H_2_O–H_2_					H_2_S–H_2_			
*T*/K	*B*_12_^cl^	*B*_12_^1qc^	*B*_12_^QFH^	*B*_12_^QFH–T^	*U*(*B*_12_^QFH–T^)	*B*_12_^cl^	*B*_12_^1qc^	*B*_12_^QFH^	*U*(*B*_12_^QFH^)
150	–51.08	–34.13	–37.86	–40.09	4.0	–54.01	–46.19	–47.30	2.8
160	–44.18	–30.46	–33.22	–34.88	3.5	–46.69	–40.14	–40.98	2.5
170	–38.42	–27.11	–29.20	–30.46	3.0	–40.46	–34.88	–35.54	2.1
180	–33.53	–24.06	–25.68	–26.65	2.7	–35.08	–30.28	–30.80	1.8
190	–29.34	–21.31	–22.58	–23.35	2.3	–30.40	–26.22	–26.64	1.6
200	–25.71	–18.82	–19.84	–20.45	2.0	–26.30	–22.61	–22.96	1.5
210	–22.54	–16.56	–17.39	–17.88	1.8	–22.67	–19.40	–19.68	1.5
220	–19.75	–14.51	–15.19	–15.60	1.6	–19.44	–16.51	–16.75	1.5
230	–17.27	–12.65	–13.21	–13.55	1.5	–16.56	–13.92	–14.12	1.5
240	–15.06	–10.96	–11.43	–11.71	1.5	–13.96	–11.56	–11.73	1.5
250	–13.07	–9.41	–9.80	–10.04	1.5	–11.61	–9.43	–9.57	1.5
260	–11.29	–7.98	–8.32	–8.52	1.5	–9.48	–7.48	–7.60	1.5
270	–9.67	–6.68	–6.97	–7.14	1.5	–7.53	–5.70	–5.80	1.5
280	–8.19	–5.48	–5.73	–5.88	1.5	–5.76	–4.06	–4.15	1.5
290	–6.85	–4.37	–4.58	–4.71	1.5	–4.13	–2.55	–2.63	1.5
300	–5.61	–3.34	–3.53	–3.64	1.5	–2.63	–1.16	–1.23	1.5
320	–3.44	–1.50	–1.65	–1.74	1.5	0.04	1.32	1.27	1.5
340	–1.58	0.09	–0.03	–0.10	1.5	2.34	3.47	3.42	1.5
360	0.02	1.47	1.38	1.33	1.5	4.33	5.34	5.30	1.5
380	1.42	2.69	2.62	2.57	1.5	6.07	6.97	6.94	1.5
400	2.64	3.77	3.71	3.67	1.5	7.60	8.42	8.39	1.5
420	3.71	4.73	4.67	4.64	1.5	8.95	9.70	9.68	1.5
440	4.67	5.58	5.54	5.51	1.5	10.16	10.85	10.83	1.5
460	5.52	6.34	6.31	6.28	1.5	11.24	11.87	11.86	1.5
480	6.28	7.03	7.00	6.98	1.5	12.21	12.80	12.78	1.5
500	6.96	7.65	7.63	7.61	1.5	13.08	13.63	13.62	1.5
520	7.58	8.22	8.19	8.18	1.5	13.87	14.38	14.37	1.5
540	8.14	8.73	8.71	8.69	1.5	14.59	15.07	15.06	1.5
560	8.65	9.19	9.17	9.16	1.5	15.25	15.69	15.68	1.5
580	9.11	9.62	9.60	9.59	1.5	15.84	16.26	16.26	1.5
600	9.53	10.01	9.99	9.99	1.5	16.39	16.79	16.78	1.5
620	9.92	10.37	10.35	10.35	1.5	16.89	17.26	17.26	1.5
640	10.28	10.69	10.68	10.68	1.5	17.35	17.71	17.70	1.5
660	10.61	11.00	10.99	10.98	1.5	17.77	18.11	18.11	1.5
680	10.91	11.28	11.27	11.26	1.5	18.17	18.49	18.48	1.5
700	11.19	11.54	11.53	11.52	1.5	18.53	18.83	18.83	1.5
750	11.80	12.10	12.10	12.09	1.5	19.32	19.59	19.59	1.5
800	12.30	12.57	12.57	12.56	1.5	19.97	20.22	20.22	1.5
850	12.72	12.97	12.96	12.96	1.5	20.52	20.74	20.74	1.5
900	13.08	13.30	13.29	13.29	1.5	20.98	21.18	21.18	1.5
1000	13.63	13.81	13.81	13.81	1.5	21.70	21.87	21.87	1.5
1100	14.03	14.19	14.18	14.18	1.5	22.21	22.36	22.36	1.5
1200	14.32	14.45	14.45	14.45	1.5	22.58	22.71	22.71	1.5
1400	14.68	14.78	14.78	14.78	1.5	23.03	23.13	23.13	1.5
1600	14.86	14.94	14.94	14.94	1.5	23.23	23.32	23.32	1.5
1800	14.92	14.99	14.99	14.99	1.5	23.29	23.36	23.36	1.5
2000	14.91	14.97	14.97	14.97	1.5	23.25	23.32	23.32	1.5

Finally, Table 2 lists the estimated combined expanded uncertainties (coverage factor *k* = 2, corresponding approximately to a 95% confidence level) of the *B*_12_^QFH–T^ values for the H_2_O–H_2_ pair and of the *B*_12_^QFH^ values for the H_2_S–H_2_ pair. The uncertainty due to the imperfections of the pair PESs stems mostly from their reduced-dimensionality treatment, i.e., the rigid-rotor approximation. Both the level of theory for the individual PES points and the quality of the analytical fits should not be significant error sources for the final *B*_12_ values. However, the semiclassical evaluation of *B*_12_ contributes to some uncertainty at the lowest temperatures. Taking these considerations and experience from previous works into account, we conservatively estimated the combined expanded uncertainties to be
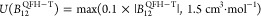
15for the H_2_O–H_2_ system and
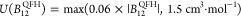
16for the H_2_S–H_2_ system.

To provide simple correlations for practical applications, we fitted polynomials in *T*^–1/2^ to the 99 calculated *B*_12_^QFH–T^ and *B*_12_^QFH^ values for the H_2_O–H_2_ and H_2_S–H_2_ systems, respectively. The polynomial structures were optimized with the Eureqa software (version 1.24.0).^47^ The resulting correlations, in which T* = *T*/(100 K), are given by
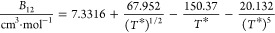
17for the H_2_O–H_2_ system and
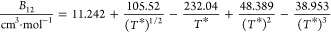
18for the H_2_S–H_2_ system. The correlations reproduce the calculated *B*_12_ values within 0.04 cm^3^·mol^–1^ and extrapolate to temperatures below 150 K and above 2000 K in a physically reasonable manner.

**Figure 7:**
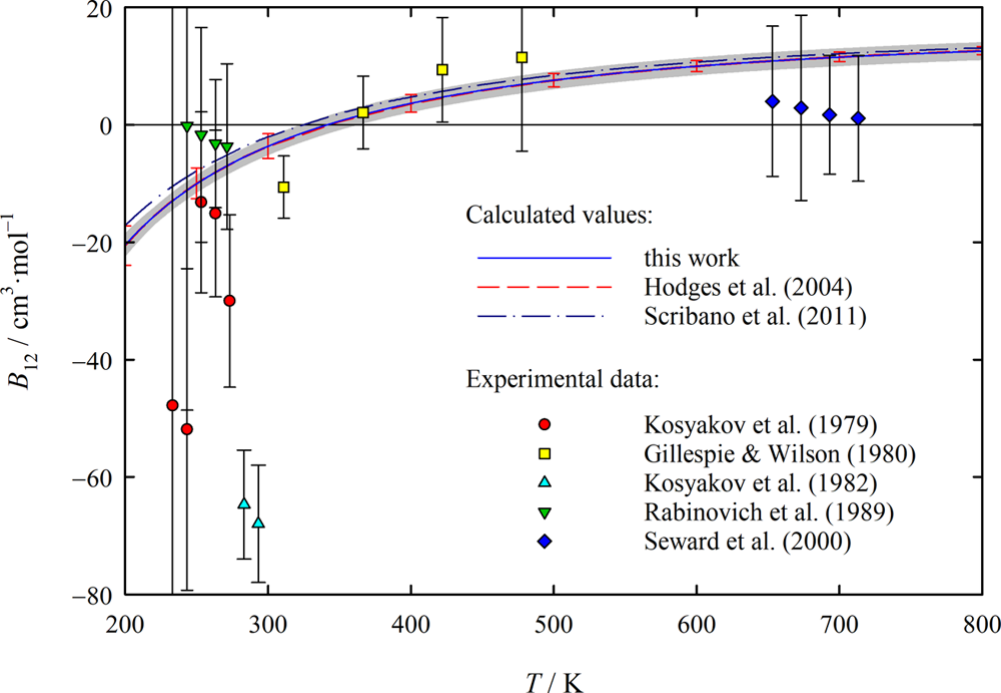
Cross second virial coefficient *B*_12_ of the H_2_O–H_2_ system as a function of temperature *T*. The calculated values of this work, for which the gray shading indicates the expanded (*k* = 2) uncertainty, correspond to the tuned QFH level of theory, while those of Hodges et al.^5^ (barely distinguishable from our values) correspond to semiclassical and, at the lowest temperatures, fully quantum-mechanical levels of theory. Scribano et al.^8^ calculated their *B*_12_ values at the 1qc level. All three sets of calculated values are based on different ab initio PESs. The experimental *B*_12_ data shown were derived by Hodges et al.^5^ from other measured quantities provided in refs (48−52).

Figure 7 depicts the *B*_12_ values for the H_2_O–H_2_ system obtained in this work at the tuned QFH level and those obtained by Hodges et al.^5^ and Scribano et al.,^8^ who each provided their results in the form of a correlation. Hodges et al.’s correlation is based on fully quantum-mechanical calculations of *B*_12_ at temperatures up to 250 K and semiclassical calculations at higher temperatures using their own ab initio PES, while Scribano et al.’s correlation is based solely on semiclassical *B*_12_ values at the 1qc level calculated using the ab initio PES of Valiron et al.^7^ The figure shows an excellent agreement between the present results and those of Hodges et al., whereas Scribano et al.’s values are somewhat higher and below ambient temperature also outside the estimated expanded uncertainty range of our values. More positive virial coefficient values indicate that the PES is on average less attractive, which is in line with the discussion in section 2.3 regarding the depths of the minima on the PESs of Valiron et al. and of this work. In contrast, the maximum well depths of the present PES and of that of Hodges et al. are very close, so that assuming that this is not a fluke, it is unsurprising that the resulting *B*_12_ values are also in excellent agreement.

**Figure 8:**
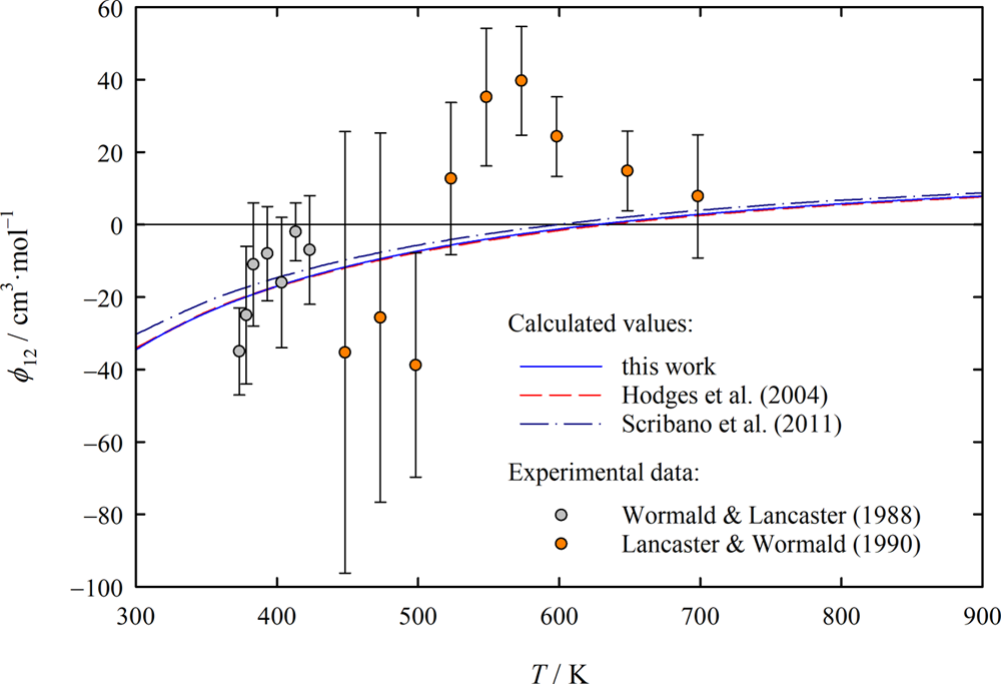
Dilute gas cross isothermal Joule–Thomson coefficient, ϕ_12_ = *B*_12_ – *T*(d*B*_12_/d*T*), of the H_2_O–H_2_ system as a function of temperature *T*. The values of this work were calculated using eq 17 and those of Hodges et al.^5^ and Scribano et al.^8^ using the correlations provided in these papers. The experimental ϕ_12_ data of Wormald and Lancaster^53^ were given in the original paper, while those of Lancaster and Wormald^55^ were derived by Hodges et al.^5^ from the measured enthalpies of mixing given in ref (55).

Apart from Wormald and Lancaster,^53^ whose *B*_12_ values we briefly discuss below in connection with Figure 8, Hodges et al.^5^ provided the only experimentally based *B*_12_ values available in the literature, which they derived from other measured quantities given in refs (48−52) and which are also shown in Figure 7. Since the scatter and the uncertainties of the data exceed the uncertainties of the calculated values substantially, the data are not suitable for verifying the calculated values. Note that we repeated Hodges et al.’s analysis of the data of Seward et al.^52^ for the second virial coefficients of different H_2_O–H_2_ mixtures using newer values for the second virial coefficient of pure H_2_,^54^ but the changes in the *B*_12_ values due to the reanalysis do not exceed 0.1 cm^3^·mol^–1^, which would not even be visible in the figure.

Figure 8 shows the dilute gas cross isothermal Joule–Thomson coefficient ϕ_12_ of the H_2_O–H_2_ system derived from the *B*_12_ correlations of this work and of Hodges et al.^5^ and Scribano et al.^8^ using the relation
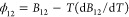
19The figure also displays the available experimental ϕ_12_ data,^53,55^ with those for ref (55) having been obtained by Hodges et al.^5^ from the measured enthalpies of mixing given in that reference. Wormald and Lancaster^53^ used their ϕ_12_ data to derive the above-mentioned *B*_12_ values that we omitted from Figure 7 because these *B*_12_ values cannot provide more information than the ϕ_12_ data they are based on. It is thus also not surprising that the pattern of the deviations for *B*_12_ mirrors that for ϕ_12_. As in the case of *B*_12_, the agreement between the present ϕ_12_ values and those of Hodges et al. is excellent, while the values for the correlation of Scribano et al. are noticeably higher and the quality of the experimental data is again insufficient for using them to assess the quality of the calculated values.

For the H_2_S–H_2_ system, to the best of our knowledge, neither *B*_12_ nor ϕ_12_ were previously calculated from first-principles or measured experimentally.

## 5 Conclusions

The cross second virial coefficients *B*_12_ of the H_2_O–H_2_ and H_2_S–H_2_ systems were determined employing a proven first-principles methodology. In the first step, new H_2_O–H_2_ and H_2_S–H_2_ pair PESs were developed using the counterpoise-corrected supermolecular approach^27^ with high-level ab initio methods up to CCSDT(Q)^37,38^ and with consideration of scalar relativistic effects. The PESs are represented analytically by site–site functions, which we provide in the form of Fortran 90 codes as part of the Supporting Information.

In the second step, we extracted *B*_12_ values for the temperature range from 150 to 2000 K from the analytical potential functions classically and with two semiclassical approaches using the MSMC technique.^45^ Our final recommended results are provided together with conservative uncertainty estimates both as a data table of discrete values and in the form of simple correlations, eqs 17 and 18, which not only represent the calculated values very accurately but also extrapolate in a reasonable manner to temperatures far below and above the investigated range.

Our *B*_12_ values for the H_2_O–H_2_ system are in excellent agreement with the first-principles values of Hodges et al.,^5^ whereas the more recent first-principles values of Scribano et al.^8^ are significantly higher. The available experimental *B*_12_ data for the H_2_O–H_2_ system are far less accurate than any of the first-principles sets of *B*_12_ values. In the case of the H_2_S–H_2_ system, we are not aware of any previous first-principles or experimental determinations of *B*_12_.

## Supporting Information Available

Further details on the internal coordinates of the H_2_O–H_2_ and H_2_S–H_2_ molecule pairs, detailed results of the ab initio calculations of the pair interaction energies and the multipole moments of H_2_O and H_2_S, the details of the distinct minimum structures obtained with the new analytical pair PESs, and Fortran 90 implementations of the PESs (ZIP)
